# Case Report: Sars-CoV-2 Infection in a Vaccinated Individual: Evaluation of the Immunological Profile and Virus Transmission Risk

**DOI:** 10.3389/fimmu.2021.708820

**Published:** 2021-06-23

**Authors:** Claudia Strafella, Valerio Caputo, Gisella Guerrera, Andrea Termine, Carlo Fabrizio, Raffaella Cascella, Mario Picozza, Carlo Caltagirone, Angelo Rossini, Maria Pia Balice, Antonino Salvia, Luca Battistini, Giovanna Borsellino, Emiliano Giardina

**Affiliations:** ^1^ Genomic Medicine Laboratory UILDM, IRCCS Santa Lucia Foundation, Rome, Italy; ^2^ Department of Biomedicine and Prevention, Tor Vergata University, Rome, Italy; ^3^ Neuroimmunology Unit, IRCCS Santa Lucia Foundation, Rome, Italy; ^4^ Department of Clinical and Behavioral Neurology, IRCCS Santa Lucia Foundation, Rome, Italy; ^5^ Medical Services, IRCCS Santa Lucia Foundation, Rome, Italy; ^6^ Clinical Microbiology Laboratory, IRCCS Santa Lucia Foundation, Rome, Italy

**Keywords:** vaccination, COVID19, immune response, Sars-CoV-2 infection, immunology

## Abstract

During the COVID19 pandemic, a range of vaccines displayed high efficacy in preventing disease, severe outcomes of infection, and mortality. However, the immunological correlates of protection, the duration of immune response, the transmission risk over time from vaccinated individuals are currently under active investigation. In this brief report, we describe the case of a vaccinated Healthcare Professional infected with a variant of Sars-CoV-2, who has been extensively investigated in order to draw a complete trajectory of infection. The patient has been monitored for the whole length of infection, assessing the temporal viral load decay, the quantification of viral RNA and subgenomic mRNA, antibodies (anti Sars-CoV-2, IgA, IgG, IgM) and cell-mediated (cytokine, B- and T-cell profiles) responses. Overall, this brief report highlights the efficacy of vaccine in preventing COVID19 disease, accelerating the recovery from infection, reducing the transmission risk, although the use of precautionary measures against Sars-CoV-2 spreading still remain critical.

## Introduction

Since the outbreak of the novel pathogenic Severe Acute Respiratory Syndrome Coronavirus 2 (Sars-CoV-2) pandemic in 2019, 128 million people have been infected across more than 200 countries (1). In addition, the extensive sequencing of Sars-CoV-2 genomes throughout the world raised the attention concerning the circulation of Sars-CoV-2 Variants of Concerns (VOCs), which have been associated with increased transmissibility and potential to escape human immune defenses (2). Many unresolved questions remain concerning the duration of immunity following natural infection, the ability of asymptomatic reinfected individuals to transmit the virus and the onset of additional VOCs in the near future (1, 2). In this context, a range of vaccines has been developed, showing high efficacy in preventing COVID19 disease, severe outcomes of infection, and mortality (3). However, the immunological correlates of protection against Sars-CoV-2 infection, the duration of the immune response, the transmission risk over time from vaccinated individuals are currently under active investigation. Clearly, T cell responses are critical for the control and the resolution of Sars-CoV-2 infection (4–6) together with the neutralizing antibody (Ab) response elicited by vaccination.

## Case Presentation

We report the case of a 38 year old-individual working as Healthcare Professional (HCP) in IRCCS Santa Lucia Foundation in Rome (Italy). The patient was vaccinated with Comirnaty vaccine from Pfizer-BioNTech (BNT162b2) (Pfizer, Inc; Philadelphia, Pennsylvania) according to the reference guidelines. The patient received her first dose on 7 January 2021 and the second dose after 21 days, as recommended. The patient participated to the anti-COVID19 routine surveillance, reporting negative results to molecular analyses until 40 days post vaccination. Serological assessment of the response to the vaccine revealed the presence of anti-Sars-CoV-2 S-protein RBD and related-subpopulations 26 days after vaccination. At 54 days from vaccination, the patient resulted to be positive to Sars-CoV-2 infection following molecular analysis of a nasopharyngeal swab-derived sample. In particular, two (namely, ORF1ab and N genes) out of three tested Sars-CoV-2 targets were detected, highlighting a high viral load and the potential infection with a variant, given the S gene-target failure. Testing for Sars-CoV-2 VOCs revealed the presence of N501Y and HV69-70del mutations within the S gene, which are associated with the VOC 202012/01 (lineage B.1.1.7) (7). Despite a high viral load and the presence of the highly transmissible and virulent variant, the patient was completely asymptomatic for COVID19 disease.

At the contact tracing, it emerged that 8 days before becoming positive the patient had participated to a sport competition in Milan as physiotherapist, after which other subjects were positive to Sars-CoV-2.

The patient was therefore subjected to close monitoring until complete resolution of infection. A comprehensive overview of the infection trajectory of the patient was drawn, considering the time length, temporal viral load decay, subgenomic-mRNA (sg-mRNA) and immune responses. For molecular analyses, 5 swab samples were taken during 10 days of follow-up, whereas 2 blood samples (on day 2 and day 9 post infection, respectively) were taken for immunological profiling. The patient provided written informed consent for this study.

## Laboratory Investigations

### Molecular Analysis and Assessment of Viral Load and Temporal Decay

Nasopharyngeal swabs were obtained using cotton swabs and viral transport media in Universal Transport Medium (UTM) (Copan Diagnostics).

Molecular diagnosis of Sars-CoV-2 infection was performed by purifying viral RNA from 300 µL of UTM through automated extraction by Magpure virus DNA/RNA purification kit (Hangzhou Bigfish Bio-tech Co. Ltd.) on Nuetraction 32 Nucleic Acid Purification System (Hangzhou Bigfish Bio-tech Co. Ltd.). The extracted RNAs were then subjected to one-step Real Time- PCR (RT-PCR) by means of TaqPath COVID-19 RT PCR CE IVD kit (ThermoFisher Scientific) using QuantStudio 5 RT-PCR system according to manufacturer’s instructions.

The results were analyzed and interpreted using the QuantStudio DA2 and Covid-19 Interpretive Softwares (ThermoFisher Scientific). Copies/mL of the viral load were determined using the Ct value related to serial dilutions of the Positive Control included in the kit.

The identification of Sars-CoV-2 variants was performed by means of RT-PCR using COVID-19 Variant Catcher CE IVD kit (Clonit). In particular, 5 µL from extracted RNA was tested using QuantStudio 5 RT-PCR system (ThermoFisher Scientific) according to manufacturer’s instructions.

The presence of subgenomic-mRNA (sg-mRNA) in extracted RNA samples was assessed by means of retrotranscription and RT-PCR following the protocol described by Wolfel et al. (8). In particular, 50 ng of extracted RNAs were tested using TaqMan assays specific for N, S and E genes retrieved from Corman et al. (9).

The evaluation of the temporal viral load decay of the patient has been performed by comparing the temporal viral load decay of a reference group matched for age (30<Age<45), gender and viral load at T0 of infection. This reference group included 122 non-vaccinated subjects (including individuals with wild-type and B.1.1.7 variant of Sars-CoV-2) sampled from a group of 1134 individuals based on their logarithmic viral load scale at Time 0 (lower bound = 3.337919 and upper bound = 5.877630). A Grubbs Test was used to assess whether the patient represented an outlier for viral load decay with respect to the reference group. The slope of a regression line representing the rate of change in viral load based on time (7± 2 days) was computed for patient and reference samples. All of the statistical analyses were performed in R version 4.0.4.

### Evaluation of Anti Sars-CoV-2 Antibodies (Abs) and Related Subpopulations

The measurement of anti Sars-CoV-2 Abs was performed by electrochemiluminescence sandwich immunoassay (ECLIA) through Roche Elecsys Anti-Sars-CoV-2 S (Roche diagnostics, Switzerland). The assay detects quantitative total Abs directed against the Receptor-Binding protein Domain (RBD) of the viral S protein. The serum was diluted 1:10 and then 1:20 with Diluent 2 Roche (Roche diagnostics, Switzerland). The neutralizing Ab were measured on cobas 601 modular analyzer (Roche diagnostics, Switzerland), using a cut-off of 0.8 U/ml to determine Abs levels. In particular, Elecsys Anti-Sars-CoV-2 S U/mL measurements are equivalent to WHO International Standard Binding Arbitrary Units per mL (BAU/mL), according to which higher values than 0.8 BAU/mL are considered positive.

Concerning the measurement of Abs subpopulations (namely IgG, IgA and IgM), the EUROIMMUN Anti-Sars-CoV-2 assay (EUROIMMUN Medizinische Labordiagnostika AG, Lübeck, Germany) was utilized. Both the IgG, IgA and the IgM ELISA tests are classic sandwich, with IgG, IgA referred to Ab anti S1 and IgM referred to Ab anti N antigen). The antibodies subpopulation was measured on Analyzer I-2p EUROIMMUN (EUROIMMUN Medizinische Labordiagnostika AG, Lübeck, Germany). Semi-quantitative results were evaluated by calculating a ratio in which the absorbance value of the controls or patient samples are related to the absorbance value of the calibrator. Ratio results <0.8 are interpreted as negative,>=0.8 to 1.1 as borderline and >=1.1 as positive.

### Assessment of Cell-Mediated Immune Responses to Sars-CoV-2 Infection

Peripheral blood mononuclear cells (PBMCs) were isolated from whole blood by density gradient centrifugation using standard procedures (Ficoll-Paque Plus, GE Healthcare). *In vitro* stimulation of antigen-specific T cells was performed with a mix of PepTivator Sars-CoV-2 peptides: nucleoprotein N, membrane protein M, and spike protein S, S1 and S+ peptide pools (Miltenyi Biotec). Each peptide was added at the final concentration of 1 mg/ml to 1,5*10^6^ PBMC cultured in RPMI 1640 complemented with pen/strep and 5% human serum, in the presence of aCD40 purified antibody (0,5 µg/ml, Miltenyi Biotech). PBMCs stimulated with Dynabeads™ Human T-Activator CD3/CD28 (Thermo Fisher Scientific) (0.5 µl/10^5^ cells) or PMA (25 ng/ml, Sigma-Aldrich) and ionomycin (200 ng/ml, Sigma-Aldrich) were used as positive control. Brefeldin A (10 µg/ml), Monensin (5µM) and anti-human CD107a antibodies were added to the cultures to allow measurement of cytokines and degranulation.

*In vitro* stimulation was performed at 37°C, 5% CO2 and after 18 hours of culture, cells were processed for combined surface and intracellular staining (see **Supplementary Table 1** for reagents used) following standard procedures, and then analyzed by flow cytometry.

Samples were acquired on a CytoFLEX flow cytometer (Beckman Coulter), equipped with three lasers. For each sample, lymphocytes were selected based on physical size (FSC) and grain size (SSC) parameters, and dead cells and doublets were excluded. The data was compensated and analyzed using FlowJo v10.7.1 (BD Biosciences).

## Results and Discussion

**Figure 1:**
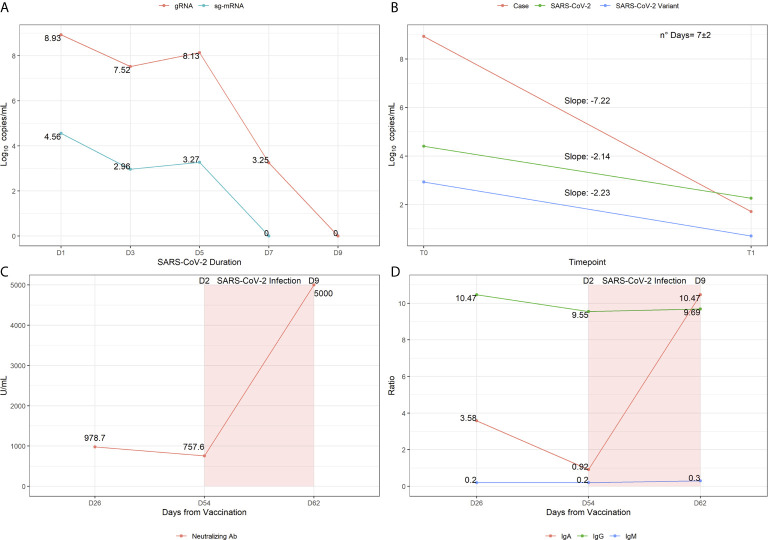
Virological and immunological profiling showing the infection trajectory observed in the patient. **(A)** Sars-CoV-2 genomic RNA and sg-mRNA component expressed in log10copies/mL are reported for each molecular test performed during the time of infection. **(B)** Slopes representation indicating the speed of temporal decay observed in the patient compared to reference samples, with wild-type or variant Sars-CoV-2. **(C)** Titers of Anti-Sars-CoV-2 RBD antibodies (i.e neutralizing Abs) measured 26 days post vaccination, 54 days post vaccination (day 2 post infection) and 62 days post vaccination (day 9 post infection). **(D)** Assessment of Antibodies subpopulations ratios measured at the three time-points of interest.

Upon close monitoring and follow-up examinations, the patient showed persistent Sars-CoV-2 viral load in the first 5 days of infection, with a pronounced decrease of viral load at day 7 (**Figure 1A**). This result suggests that the patient would have been able to transmit the virus only in the first 5 days of infection because of the persistent viral load. Supporting this hypothesis, the sg-mRNA was assessed since it may indicate the effective rate of infectiousness of patients (8). Indeed, the quantification of sg-mRNA in the 5 nasopharyngeal swab samples revealed a detectable signal only in the first 5 days of infection (**Figure 1A**), consistent with the high viral load detected for the overall Sars-CoV-2 genome.

Subsequently, the unusual temporal viral load decay observed in this patient was compared to that of reference samples of non-vaccinated individuals (n=122). Statistical analysis revealed that the patient represents an outlier in terms of viral load (*p*=0.00017) and speed of viral decay (*p*=0.0047), indicating that the patient took less time to resolve the infection compared to the reference non-vaccinated group (visualized as slope distribution in **Figure 1B**).

Concerning the Ab response to infection, the analysis of anti Sars-CoV-2 Ab at day 2 post infection (p.i.) (54 days post vaccination, p.v.) revealed a slight decrease of anti-RBD Ab levels (**Figure 1C**) and a sharp decrease of IgA with unaltered IgG and IgM subpopulations compared to previous titers (**Figure 1D**). At day 9 p.i. (62 days p.v.), a substantial increase of anti-RBD Abs and IgA was detected, whereas total IgG and IgM levels were comparable to the previous measurements (**Figures 1C, D**). Although there is a significant interindividual difference in the levels of neutralizing Abs, the great increase of anti-RBD Abs and of IgA p.i. are suggestive of an effective activity of the vaccine and may underlie the fast recovery from infection. In particular, the results concerning IgA are very intriguing given their crucial role in the protection from infection. Although this role has not yet been finally defined, recent studies showed that the appearance of these secretory immunoglobulins may contribute to the effective viral neutralization at the mucosal surface of the respiratory system and, thereby, to the prevention of viral spreading (10).

**Figure 2:**
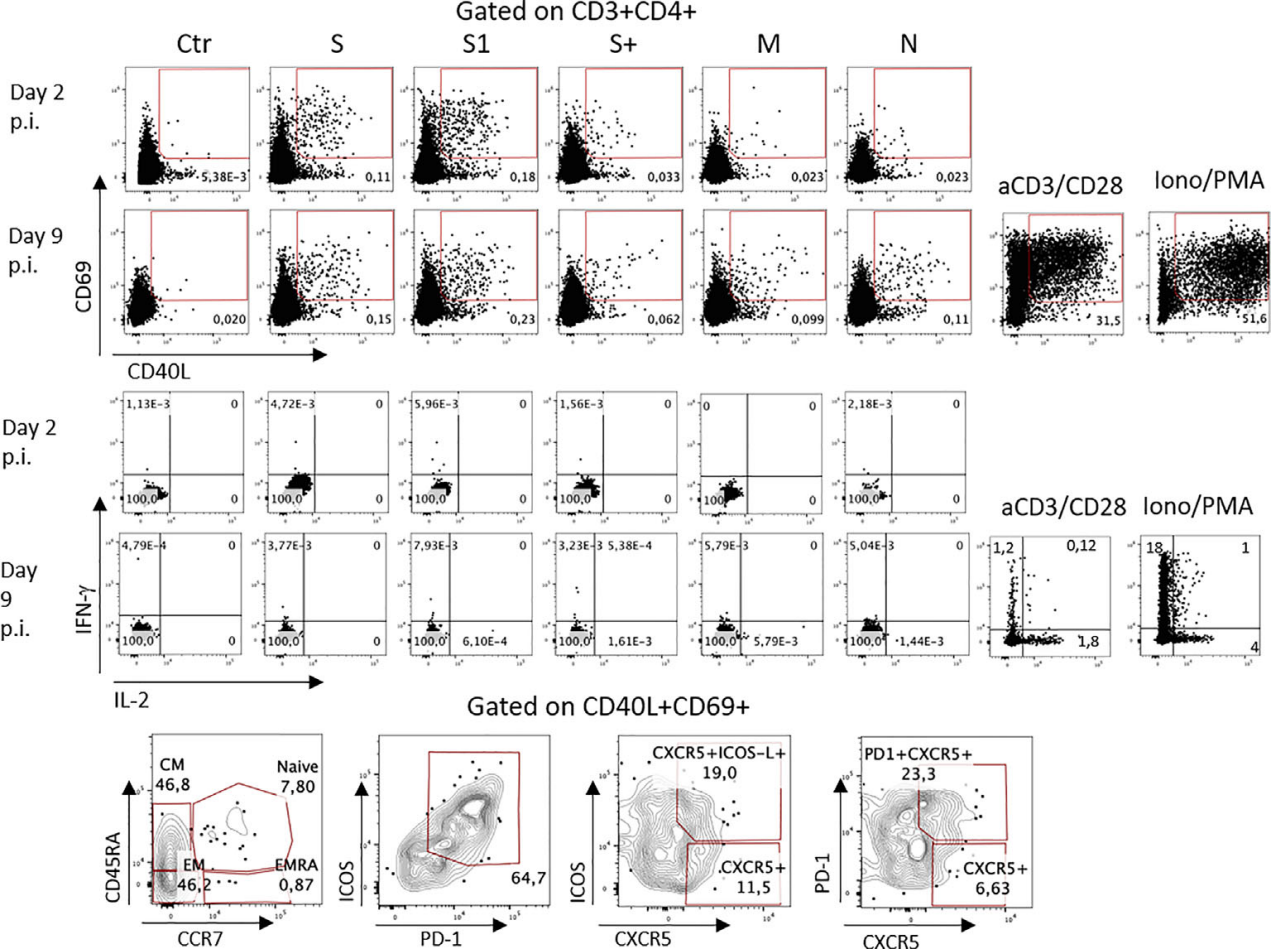
SARS-CoV2-specific CD4 T cell responses. (1) Surface expression of activation markers CD69 and CD40L by CD4 cells stimulated with peptide pools from the different viral antigens. Numbers indicate percentages of positive cells within each gate. (2) IFN-γ and IL-2 production by antigen-specific CD4 cells. Bottom: phenotype of S-specific CD4+ T cells. Ab: Antibodies. p.i: post infection.

Furthermore, an immunological profiling of peripheral lymphocytes was performed on days 2 and 9 p.i. by high-parameter flow cytometry. Sars-CoV-2-specific T cells were identified by assessing the upregulation of surface activation-induced markers following cell culture with Sars-CoV-2-derived overlapping peptide pools of the different viral proteins. At day 2 p.i. S protein-specific CD4+ T cells were detected through their upregulation of CD40L and CD69 (**Figure 2**, upper panels), while M and N protein-specific cells were barely detectable, as expected in an individual vaccinated with Cominarty (encoding only the viral Spike protein). The fraction of spike-specific CD4+ T cells increased at day 9 p.i., together with N- and M-protein reactivity. A closer look at these antigen-specific cells revealed a differentiated effector- and central-memory phenotype, with most cells expressing PD-1, ICOS, and CXCR5, indicating differentiation in follicular helper cells able to sustain B cell maturation and Ab production. However, these cells did not produce neither IFN-γ nor IL-2, suggesting a limp direct antiviral function although stimulation with polyclonal stimuli elicited a normal response. In addition, CD8+ T cells responded poorly to SARS-CoV2 peptides, with limited upregulation of activation markers (CD69 and CD137) and minimal cytotoxic degranulation (measured by CD107a expression) and IFN-γ production (**Supplementary Figure 1**). The high viral load detected in this individual may be due, at least in part, to the feeble direct antiviral response by both CD4 and CD8 T cells, with inefficient contrast to viral replication. However, within B cells, a significant fraction of plasmablasts was identified at day 2 p.i denoting that these cells were activated by the infection and were appropriately differentiating in antibody-secreting cells, likely supported by the CD4 helper T cells. This correlated with the rapidly increasing anti-RBD Abs levels found in the serum on day 9.

Although this subject was infected with a high viral load, the immediate immune response induced by vaccination quickly and efficiently controlled the viral spread preventing disease and reduced transmission risk thanks to a lower time of infectiousness and a faster clearance of the viral infection. This result is consistent with recent literature data showing that vaccinated individuals infected with Sars-CoV-2 are mostly asymptomatic, rapidly recover from infection and show reduced viral transmission risk (11, 12). However, vaccinated subjects investigated in these studies showed lower viral loads compared to the patient of this study, although very few data are available on this topic (11, 12).

An effective adaptive immune response involves the combined action of B lymphocytes, CD4 T cells, and cytotoxic CD8 T cells (13). However, successful clearance of infections can also occur in case of defective or lagging function of one of these components. Our results show that in this individual, high Ab titers and CD4 T cell helper were able to control the viral infection in a few days, although vaccination did not induce sterilizing immunity. The study of the effector function (cytokine profile and cytotoxicity) of T cells could help in predicting the extent of immune protection in vaccinated individuals.

## Conclusions

Overall, this case report confirms that the vaccine prevents severe forms of COVID19 disease/mortality, accelerates the resolution of viral infection and reduces transmission risk by decreasing the time of infectiousness. Moreover, it emphasizes the importance of paying careful attention to the risk of transmission from asymptomatic vaccinated individuals by maintaining the precautionary measures for preventing Sars-CoV-2 spread. This aspect should be especially undertaken within the healthcare working places by maintaining the periodical surveillance of the HCPs. Finally, although this case report refers to a single patient, and further studies are necessary to better understand the immune response to vaccine and its related impact on transmission risk, we highlight the finding of an incomplete T cell response to the infection, which may underlie the transitory high viral load observed in this individual despite vaccination.

## Data Availability Statement

The original contributions presented in the study are included in the article/**Supplementary Material**. Further inquiries can be directed to the corresponding author.

## Ethics Statement

Ethical review and approval was not required for the study on human participants in accordance with the local legislation and institutional requirements. The patients/participants provided their written informed consent to participate in this study.

## Author Contributions

Conception and design of the study: CS, VC, GG, LB, GB, and EG. Acquisition, analysis and interpretation of data: CS, VC, GG, AT, CF, RC, MP, CC, AR, MB, AS, LB, GB, and EG. Statistical analysis: AT and CF. Writing of the manuscript draft: CS, VC, GG, AR, AS, GB, and EG. Critical revision of the manuscript: RC, MP, CC, AR, MB, AS, LB, GB, and EG. All authors contributed to the article and approved the submitted version.

## Funding

The work has been partially supported by the Italian Ministry of Health (project COVID-2020-12371735) to LB.

## Conflict of Interest

The authors declare that the research was conducted in the absence of any commercial or financial relationships that could be construed as a potential conflict of interest.
