# Supplementary material for: Case Report: Sars-CoV-2 Infection in a Vaccinated Individual: Evaluation of the Immunological Profile and Virus Transmission Risk
Source: Front Immunol. 2021 Jun 23;12:708820. doi: 10.3389/fimmu.2021.708820 (PMC8270685; doi:10.3389/fimmu.2021.708820)
Supplement: Supplementary file 1 [file DataSheet_1.docx]

Supplementary Material

**Supplementary Table 1: Reagents used for surface and intracellular staining.**

| Antibody | Fluorochrome | Company | Clone | Titer |
| --- | --- | --- | --- | --- |
| CD3 | BUV496 | Bect.Dick. | UCHT1 | 1:100 |
| CD4 | iFluor810 | AAT Bioquest | RPA-T4 | 1:200 |
| CD8 | iFluor594 | AAT Bioquest | SK1 | 1:150 |
| CD19 | BV650 | Bect. Dick. | SJ25C1 | 1:40 |
| CD45RA | BV480 | Bect. Dick. | HI100 | 1:200 |
| CD69 | BB700 | Bect. Dick. | FN50 | 1:100 |
| CD107a/LAMP-1 | BV421 | Bect. Dick. | H4A3 | 1ul/well 10^6cells |
| CD137 | BUV395 | Bect. Dick. | 4B4-1 | 1:30 |
| CD154 (CD40L) | PE | eBioscience | 24-31 | 1:30 |
| CD185 (CXCR5) | APC-R700 | Bect. Dick. | RF8B2 | 1:60 |
| CD278 (ICOS) | APC | Coulter | ISA-3 | 1:100 |
| CD279 (PD-1) | BV421 | Bect. Dick. | EH12.1 | 1:50 |
| CCR7 | PE-Cy7 | SONY | G043H7 | 1:30 |
| HLA-DR | APC-Vio770 | Miltenyi | REA805 | 1:120 |
| HLA-DR | BV785 | Bect. Dick. | G46-6 | 1:100 |
| IFNg | APC | Bect. Dick. | B27 | 1:100 |
| IL-2 | PE-CF594 | Bect. Dick. | 5344.111 | 1:50 |
| Live Dead | Promo Fluor 840 | Promokine |  | 1:10.000 |

**
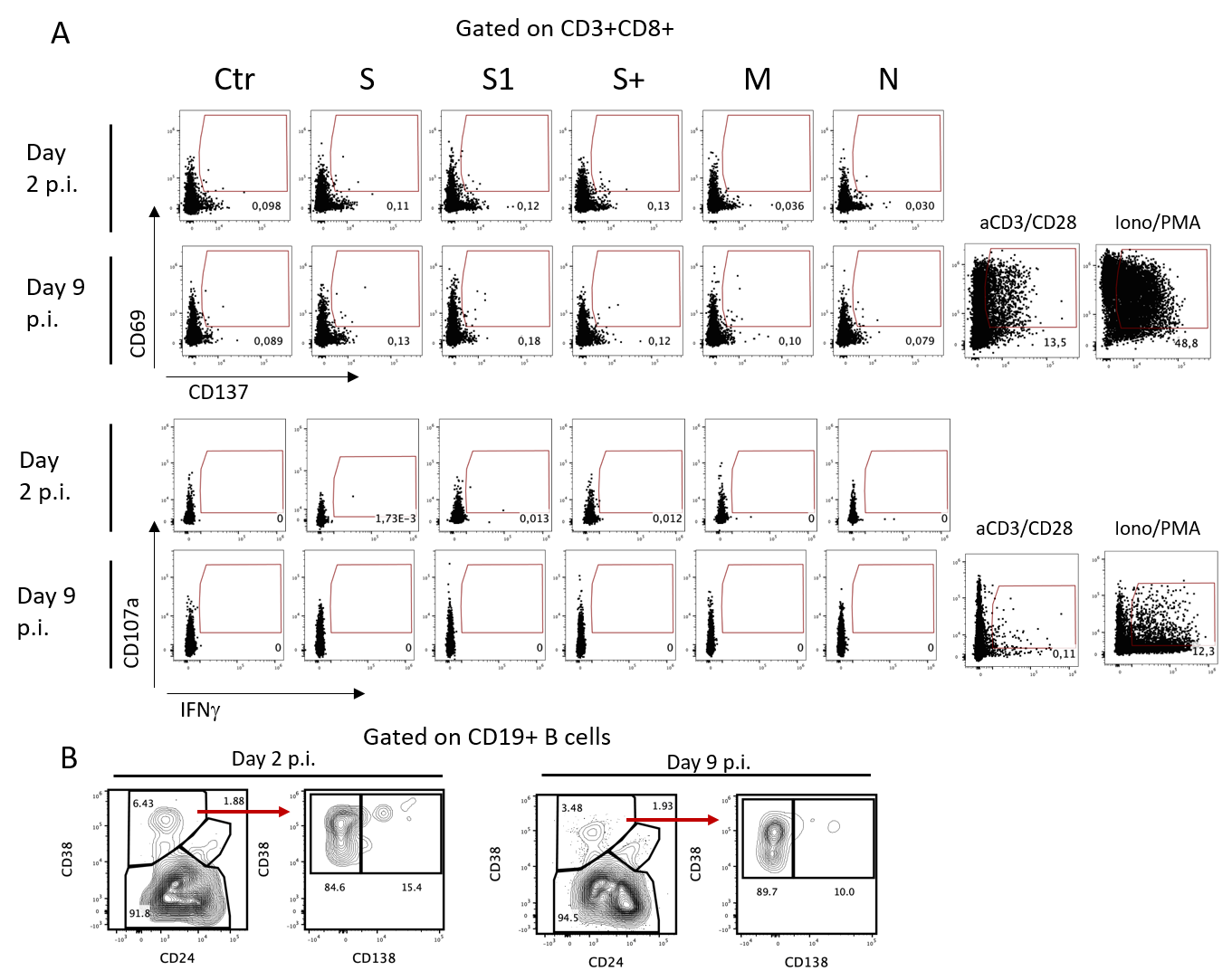
**

**Supplementary Figure 1: SARS-CoV2-specific CD8 T cell responses.** Fresh PBMCs were incubated o.n. with overlapping peptides from the different viral proteins. (A) Top panels: CD8 T cell activation was detected through measurement of CD137 and CD69 expression at day 2 and day 9-post infection. Lower panels: measurement of degranulation (CD107a) and IFN-γ production by CD8 T cells stimulated with the peptides. (B) Increased frequency of plasmablasts (CD38highCD24-) and detection of plasmacells (CD38highCD138+) in the first days post infection. p.i.: post infection.
